# Exploring the perspectives of antimicrobial stewardship pharmacists in England on the subscription model for antimicrobial drugs

**DOI:** 10.1093/jacamr/dlag018

**Published:** 2026-02-17

**Authors:** Stephen Hughes, Ioannis Baltas, Mark Gilchrist

**Affiliations:** Pharmacy, Chelsea & Westminster NHS Foundation Trust, London SW10 9NH, UK; Infection, Immunity & Inflammation Department, UCL Great Ormond Street Institute of Child Health, London WC1N 1EH, UK; British Society for Antimicrobial Chemotherapy, 53 Regent Place, Birmingham B1 3NJ, UK; British Society for Antimicrobial Chemotherapy, 53 Regent Place, Birmingham B1 3NJ, UK; Department of Pharmacy/Infection, Imperial College Healthcare NHS Trust, London W2 1NY, UK; Department of Infectious Disease, Imperial College London, Exhibition Road, London SW7 2AZ, UK

## Abstract

**Background:**

The National Institute for Health and Care Excellence (NICE) introduced the world's first ‘subscription-type’ antimicrobial payment model in the UK in 2022 for ceftazidime-avibactam and cefiderocol, decoupling reimbursement from usage to stimulate innovation. The model is being expanded to additional antimicrobials. We aimed to explore the views of lead antimicrobial stewardship (AMS) pharmacists, key model implementers and stakeholders, using a nationwide survey.

**Methods:**

An online survey was distributed to lead AMS pharmacists of NHS acute Trusts (*n* = 131) in England (February–July 2025).

**Results:**

Forty-three pharmacists (response rate 32.8%) participated. Most were familiar with the model (86.1%) and supported its principle (88.4%), believing it promotes stewardship and innovation. Strong support existed for model expansion to antifungals, antivirals and novel modalities such as phage therapy.

Key priority pathogens for candidate drugs included carbapenem-resistant *Pseudomonas aeruginosa*, carbapenem-resistant *Acinetobacter baumannii* and carbapenem-resistant *Enterobacterales*. Respondents prioritized candidate drugs with oral or once-daily intravenous administration. Over half (51.2%) favoured empirical use in high-risk patients, the remainder would restrict novel antimicrobials to microbiologically-proven infections. Top risk factors for empirical use included previous infection/colonization with carbapenem-resistant bacteria and carbapenem treatment failure.

Aseptic preparation requirements were the major practical barrier, rather than drug monitoring, storage or interaction management. Median fair price for Trust contribution was 50% of the list cost.

**Conclusions:**

AMS pharmacists view the subscription model positively but highlight the need to refine product award criteria to reflect clinical implementation priorities. Their insights are critical to optimizing future delinked payment models and ensuring equitable access to novel antimicrobials.

## Introduction

Antimicrobial resistance (AMR) poses a significant threat to global health, prompting the need for innovative solutions to optimize treatment outcomes for patients with drug-resistant infections.^1^ The development of new antimicrobials is crucial in this fight.^2,3^ However, it is well documented that the traditional market model fails to provide sufficient incentives for the development of new antimicrobials, as the very stewardship practices necessary to preserve these novel therapies, reserving them for cases where older drugs have failed, ultimately limit their commercial use and profitability.^4–8^

In response to this challenge, the National Institute for Health and Care Excellence (NICE) in the United Kingdom introduced a pioneering subscription model in 2022.^7,9,10^ This novel approach aimed to create a sustainable framework for antimicrobial reimbursement and development by delinking the cost of new antimicrobials from their usage. The model was designed to align financial incentives with public health outcomes, thereby promoting antimicrobial stewardship and encouraging the creation of new treatment options.^4,5,11^

Since its introduction, the NICE subscription model has been a subject of extensive scrutiny and debate.^7^ The model pilot launched in July 2022 initially included two new antimicrobial drugs [cefiderocol (Fetroja; Shionogi) and ceftazidime-avibactam (Zavicefta; Pfizer)] and marked a significant step towards innovative drug funding.^12,13^ However, the journey has not been without hurdles. While the formal framework has helped to reduce barriers to usage, it has not entirely eliminated them. The model introduced additional administrative burdens, such as the requirement to complete Blueteq forms, an NHS digital tool used for prior authorization to ensure high-cost medicines met strict clinical criteria (a requirement that ceased in July 2025). Furthermore, the financial pressure remains significant, with NHS Trusts paying over 50% of the list price, a substantial outlay compared to older, generic treatments.^10^ Currently, NICE is rolling out the second phase of the subscription model, which will include additional novel antimicrobials, selected using a point-based system with predefined award criteria.^9^ Ongoing evaluation of the subscription model from its end users remains a crucial research priority for optimizing this innovative funding framework.

Previous studies surveyed infectious diseases and microbiology consultants during the model's initial rollout, documenting general support for the initiative.^14^ Several years on, another survey has been undertaken looking at a critical stakeholder group: antimicrobial stewardship (AMS) pharmacists, who manage the day-to-day implementation of the subscription model and navigate its practical challenges at the hospital level. This study aimed to gather insights from AMS pharmacists on the NICE subscription model's impact and its future potential, as well as inform future subscription candidate selection and Product Award Criteria.

## Materials and methods

### Study population

The target population for the SMASH II (Subscription models for antimicrobials in hospitals II) survey was all lead AMS/infection pharmacists working in NHS acute hospitals in the UK. The survey was shared through professional communications channels to ensure broad reach and participation. Target participants were contacted up to three times via email and invited to complete the study questionnaire. Responses were not anonymous, as participants provided their professional e-mail addresses with their responses to ensure data authenticity. Only fully completed responses were recorded. A financial incentive (British Society for Antimicrobial Chemotherapy conference attendance fee, worth up to £200) was offered to all participants completing the study questionnaire.

### Questionnaire design

The questionnaire was developed by the study authors (IB & MG) based on a previous study,^14^ with piloting with a selection of 10 AMS pharmacists to ensure relevance and comprehensiveness. The final version of the questionnaire is available in Appendix I (available as Supplementary data at *JAC-AMR* Online) (available as Supplementary data). The survey was administered using the online SurveyMonkey platform (Momentive Inc., USA).

### Statistical analysis

Analysis was performed in SPSS version 29 (IBM Corp., Armonk, NY, USA). Categorical variables were expressed as percentages with 95% CIs, and continuous variables as means with 95% CIs or medians with interquartile ranges (IQRs), as appropriate. For Likert scales, agreement with the question was defined as the sum of participants who selected ‘agree’ or ‘strongly agree’ for their answers.

### Ethics

Ethical approval for this study was provided by the Research Ethics Committee of the London School of Hygiene and Tropical Medicine (Ref. No. 28161/RR/29296).

## Results

A total of 43 lead antimicrobial pharmacists completed the survey between February 25 and July 25; this is a response rate of 32.8% based on the number of NHS Acute Trusts in England (*n* = 131). The median number of years of experience working as antimicrobial stewardship/infection pharmacist was 11 (IQR 7–16). Responses were limited to NHS England regions only; no responses were received from any of the three devolved nations (Scotland, Wales and Northern Ireland), where the subscription model has not yet been rolled out. London (30.2%, 13/43) was the best represented within the analysis. Only 7% (3/43) of participants reported conflicts of interest when completing the study. These responses were not excluded from the main analysis, as sensitivity analysis showed no difference in the direction and magnitude of results for all study questions when their responses were excluded (data not shown).

### General views on the subscription-type model

The majority of responding specialist pharmacists (86.1%; 37/43) had heard of the ‘subscription-type’ funding model at the time of the study; 79.1% (34/43) believed they had a good understanding of the model. Participants were asked about their level of information received at the launch of the ‘subscription-type’ payment model in July 2022; 44.2% (19/43) of respondents either disagreed or strongly disagreed with the statement that they had received adequate information about the model at launch. Only 41.9% (18/43) of respondents stated that adequate information was provided to them at launch. When asked about their views of the model’s positive impact on management of drug-resistant infections, 88.4% (38/43) of respondents either strongly agreed or agreed with the statement, indicating a high level of support among specialist pharmacists. A similar level of support was seen as pharmacists agreed the model would stimulate future research and development options in this therapeutic area (86.1%; 37/43). Many AMS pharmacists (67.4%, 29/43) agreed that cost is a significant consideration for them when using/recommending the use of specific antimicrobials, but some pharmacists (16.3%, 7/43) also disagreed with this statement.

**Figure 1.**
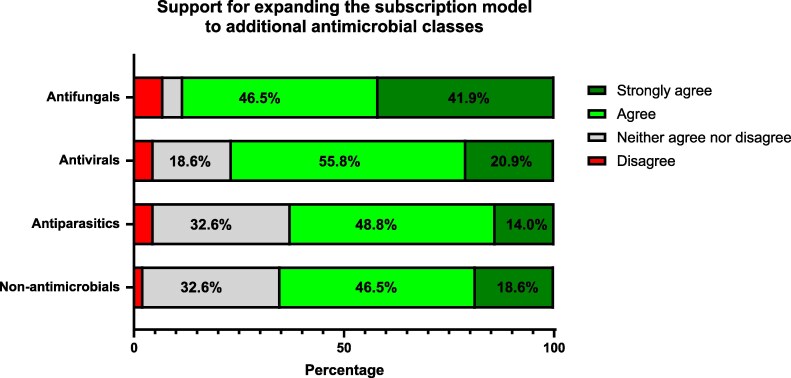
Respondents’ views on whether the UK ‘subscription-type’ payment model should be expanded to cover additional therapeutic areas (non-antimicrobial = monoclonal antibodies, phages, anti-virulence agents); (*n* = 43).

Participants were asked about their views on expanding the ‘subscription-type’ payment model to include different types of drugs. A high level of support for expanding the subscription model to include antifungals, antivirals, antiparasitics and non-antimicrobials (e.g. phage, monoclonal antibodies and anti-virulence agents) (Figure 1.).

### Use of antimicrobials through subscription model

**Figure 2.**
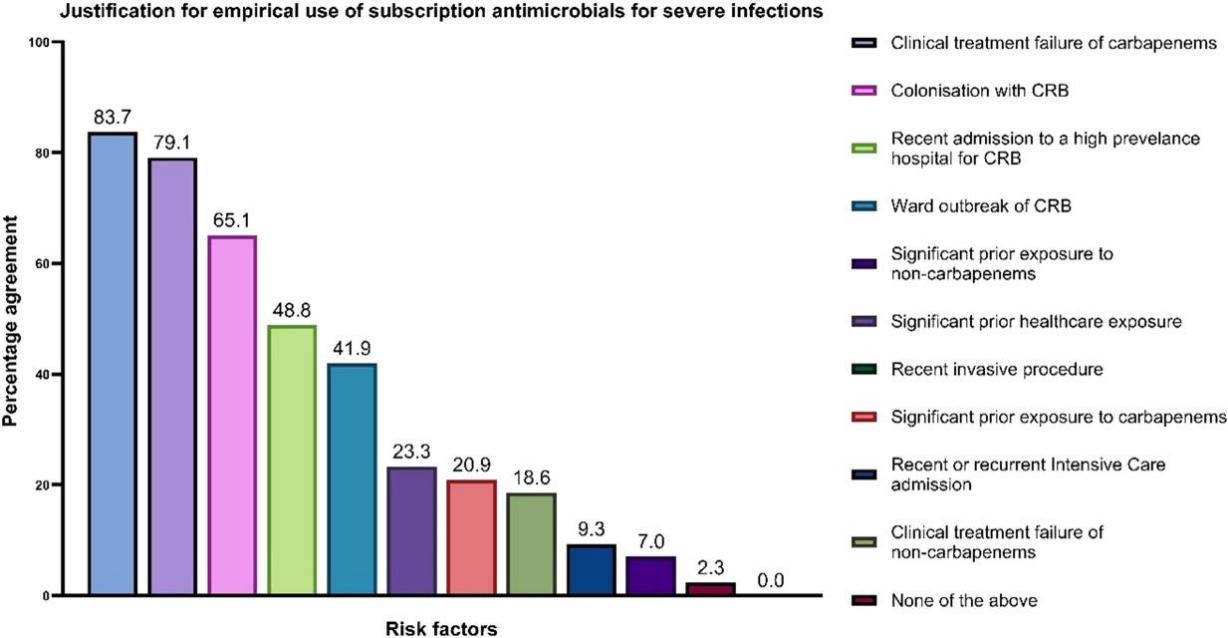
Percentage agreement on scenarios justifying empirical antimicrobial use. This bar chart displays the percentage of respondents who agreed that various risk factors would justify the empirical use of antimicrobials introduced via a ‘subscription-type’ payment model (*n* = 43; 95% CI). CRB, carbapenem-resistant bacteria.

Participants were asked to select all scenarios that would justify the empirical use of antimicrobials introduced through the ‘subscription-type’ payment model in an unwell patient with a severe infection. Patients most likely to benefit from treatment include those with previous infection with a carbapenem-resistant bacteria (83.7%, 95% CI [72.7,94.8]), clinical failure of carbapenems (79.1%, 95% CI [66.9%,91.2%]) and current colonization with carbapenem-resistant bacteria (65.1%, 95% CI [50.9%,79.4%]), Figure 2.

A follow-up question asked respondents to rank the appropriate level of restriction to be placed upon prescribing of these new antimicrobials (Table 1). Half of respondents (51.2%, 22/43) favoured the empiric use of new antimicrobials when risk factors for resistance were present without the need for microbiologically proven infection. The remaining respondents preferred to restrict usage until a microbiologically confirmed carbapenem-resistant infection was present. No respondents selected the least restrictive empirical use scenario.

**Table 1.** Participant opinion on the most appropriate clinical scenario for using new antimicrobials introduced via the subscription-type model (*n* = 43)

In your opinion, antimicrobials introduced through the ‘subscription-type’ payment model should be available as a treatment option for which of following hierarchical scenarios?	Response rate
Empirically, when there is urgent clinical need to treat an unwell patient with a severe infection, in the absence of risk factors for antimicrobial resistance, and all the scenarios below.	0.0%
Empirically, when there is urgent clinical need to treat an unwell patient with a severe infection, when risk factors for antimicrobial resistance are present, and all the scenarios below.	51.2%
In microbiologically confirmed carbapenem-resistant infections, when microbiological susceptibility and/or genetic testing for the proposed antimicrobial is unknown, and the scenario below.	32.6%
In microbiologically confirmed carbapenem-resistant infections, when microbiological susceptibility and/or genetic testing has confirmed that the infection is susceptible to the proposed antimicrobial.	16.3%

The presence of kidney dysfunction (severe or end-stage) was considered an important factor for considering early initiation with new antimicrobials. Overall, 62.79% (27/43) of respondents either agreed or strongly agreed that new antimicrobials are preferable to colistin, aminoglycosides or glycopeptides as initial treatment options due to the risks of nephrotoxicity, despite the need to restrict the use of antimicrobials introduced through the ‘subscription-type’ payment model; 23.3% disagreed with this statement.

### Off-licensed use of novel antimicrobials in vulnerable patient groups

Participants were asked to consider the appropriateness of using newly licensed antimicrobials for off-licensed indications. There was near universal agreement that these therapies should be considered for both paediatrics (90.7%; 39/43) and pregnant patients (88.4%, 38/43), respectively, for management of infections due to multidrug-resistant bacteria.

### Future of subscription model

**Figure 3.**
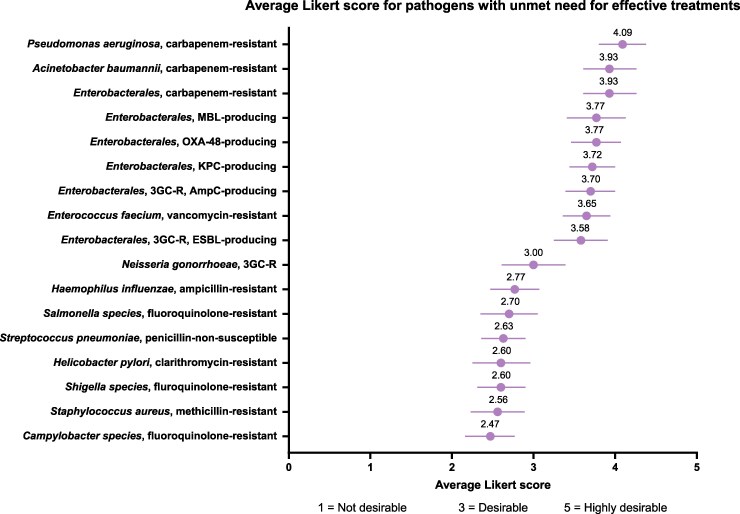
Mean Likert Score and 95% CI for problem pathogens; *n* = 43. MBL, metallo-β-lactamase; ESBL, extended spectrum β-lactamase; 3GC, third generation cephalosporin; AMP, ampicillin; PEN, penicillin.

The surveyed pharmacists were asked about the future of the ‘subscription-type’ model and factors that may influence future drug choice. Most (83.7%, 36/43) agreed that prioritization of novel antimicrobials with activity against the WHO priority pathogen list. Additionally, pharmacists were asked to assess the degree of unmet need for effective treatments for a range of pathogens on a scale of 1 to 5, with 1 indicating low unmet need and 5 indicating high unmet need, Figure 3. The pathogens with the highest weighted average scores, indicating a higher unmet need, include carbapenem-resistant *Pseudomonas aeruginosa* (median score 4.1), carbapenem-resistant *Acinetobacter baumannii* (3.9) and carbapenem-resistant *Enterobacterales* (3.9). In contrast, pathogens such as methicillin-resistant *Staphylococcus aureus* and fluoroquinolone-resistant Campylobacter species were perceived to have a lower unmet need, with scores of 2.6 and 2.5, respectively (Figure 3).

**Figure 4.**
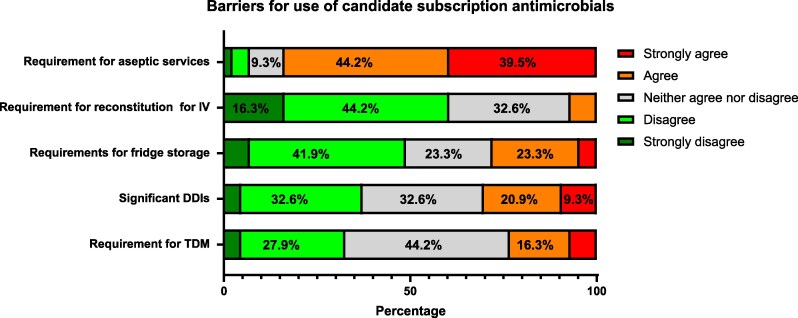
Participant rating of negative factors associated with antimicrobials; *n* = 43. IV, intravenous; DDI, drug–drug interaction; TDM, therapeutic drug monitoring.

The importance of factors such as drug–drug interactions, adverse effect profile, need for therapeutic drug monitoring, storage and reconstitution requirements, as viewed by the specialist pharmacists were also surveyed. The need for fridge storage or reconstitution of intravenous doses was not thought to be problematic for respondents (Figure 4.). The need for therapeutic drug monitoring or the presence of potent drug–drug interactions was also received with mixed views; less than 1 in 3 respondents agreed that these would be a significant problem in practice. However, the need for sterile or aseptic dose preparation was strongly agreed (83.7%, 36/43) as a significant limitation for an antimicrobial.

**Figure 5.**
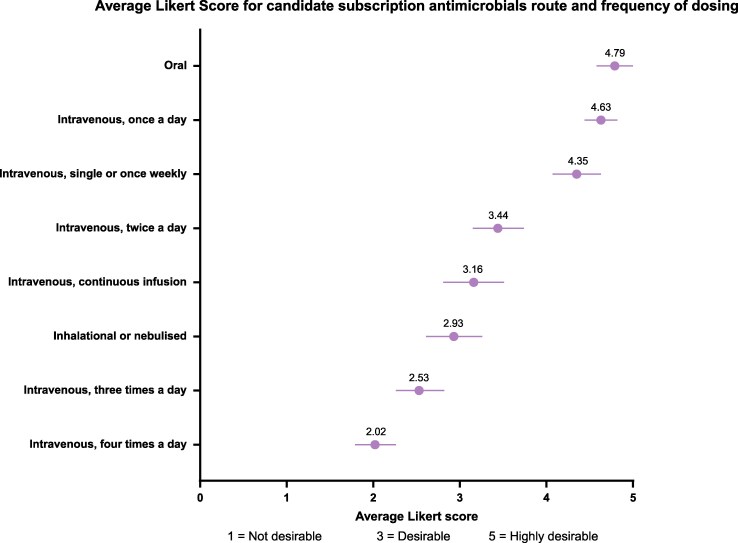
Mean Likert Score and 95% CI for desirability of routes and frequencies of administration of antimicrobials; *n* = 43.

Orally administered or a once-daily dosed intravenous infusion were the most desirable options for route and frequency of administration (Figure 5.). This interest in a once-daily intravenous option is reflected in the near total agreement (88.4%, 38/43) that future antimicrobials selected for the ‘subscription-type’ model should be suitable for administration through Outpatient Parenteral Antimicrobial Therapy (OPAT) services. Respondents agreed that the availability of oral or OPAT-friendly treatment options through the subscription model would reduce overall hospitalization and length of stay (90.7%; 39/43).

### Monitoring the outcomes and stewardship of new antimicrobials

The specialist pharmacists were asked about the need to maintain a national registry to monitor the outcomes of patients treated with new antimicrobials; 79.1% (34/43) agreed or strongly agreed that this was needed. A more mixed view was shared on the need for reporting of antimicrobial usage through a central portal (Blueteq). This optional data collection tool was in place in NHS England hospitals at the time of the survey; 37.2% (16/43) agreed or strongly agreed with the principle of Blueteq form completion. Conversely, 39.5% (17/43) disagreed or strongly disagreed with the need or support of the Blueteq forms.

### Cost impact of subscription model

Participants were asked to determine a fair price for individual Trusts to pay for a hypothetical drug with a list price of £1000 under the subscription-type payment model. Responses varied, indicating a diverse range of perspectives on what is considered fair, as shown in Table 2.

**Table 2.** Fair price determination for individual trusts (as total of £1000 drug cost)

Trust price considered fair (in £)	Number of responses
≤£100	4
£101–200	1
£201–300	5
£301–400	9
£401–500	19
£501–600	1
£601–700	1
£701–800	3
£801–900	0
>900	0

## Discussion

This survey of antimicrobial stewardship and AMS pharmacists in England provides timely insights into the utility and acceptability of the NICE subscription model since its introduction. Our findings confirm overall support from this specialist group, with most respondents agreeing that the availability of ceftazidime-avibactam and cefiderocol has positively impacted the management of patients with multidrug-resistant infections. Surveyed pharmacists expressed strong interest in expanding the subscription model beyond antibacterials to include antifungals, antivirals and even less traditional interventions such as phage therapy.

The majority of respondents support the use of novel antimicrobials in patients with known colonization or prior infection with carbapenem-resistant organisms, aligning with priorities identified among infectious diseases and microbiology consultants in an earlier 2022 survey.^14^ However, unlike their medical colleagues, specialist pharmacists demonstrated lower support for empirical use during carbapenem-resistant organism outbreaks. This divergence may reflect a more conservative stewardship-oriented approach.

Clinical use of novel antimicrobials outside of their licensed indication was widely supported by specialist pharmacists surveyed. There was high agreement reported that these agents could and should be used in high-risk patient groups such as pregnant women and paediatrics, despite the lack of real-world evidence. Most pharmacists would prioritize novel antimicrobials in patients with comorbid conditions to avoid known toxicities associated with alternative treatments, such as nephrotoxicity with colistin. This pragmatic approach to off-label use mirrors findings reported by medical colleagues regarding paediatric practice and reflects the reality of managing life-threatening infections where treatment options are severely limited.

Looking forward, we explored pharmacist perspectives on value-based assessment measures for future novel antimicrobials under the proposed ‘Product Award Criteria.’^9,15^ This framework quantifies the value of novel antimicrobials based on three components: (1) relative effectiveness and unmet clinical need, (2) pharmacological benefit, and (3) health system benefit. When assessing unmet clinical need, surveyed pharmacists prioritized three main pathogen groups for future subscription models: carbapenem-resistant *Pseudomonas aeruginosa*, carbapenem-resistant *Enterobacterales* and carbapenem-resistant *Acinetobacter baumannii*. Low prioritization was reported for methicillin-resistant *Staphylococcus aureus* despite the known clinical burden of infection within the NHS; this may reflect the respondents’ confidence in currently available therapies such as glycopeptides, lipopeptides, oxazolidinones and anti-MRSA beta-lactams. These priorities align closely with the World Health Organization's critical priority pathogens list and the value ratings proposed in the NHS England Product Award Criteria, providing reassuring concordance between expert clinical opinion and formalized priority-setting frameworks.^3^

However, notable discordance emerged between the Product Award Criteria ‘health system factors’ and pharmacist perceptions of practical value. Oral and once-daily intravenous formulations were strongly favoured for their convenience and value-adding properties, particularly their potential to facilitate outpatient management of complex infections. Current options for outpatient parenteral antimicrobial therapy (OPAT) are extremely limited for patients with multidrug-resistant infections, especially for Gram-negative pathogens, making new agents suitable for outpatient use particularly valuable. Conversely, our results showed that the use of more frequent dosing was considered less desirable (receiving progressively lower average utility scores of 3.4 for twice-daily, 2.5 for thrice-daily and 2.0 for four-times-daily administration). This pattern of weighting (favouring less frequent dosing) aligns with the Product Award Criteria, which assigns progressively lower value to more frequent dosing schedules. However, in our view, in the hospitalized population, the incremental benefit of a twice-daily over a four-times-daily administration is often minimal, questioning whether the Product Award Criteria appropriately weights this factor for the intended patient group.

Similarly, the Product Award Criteria assigns added value to drugs with minimal drug–drug interactions, adverse drug reactions, need for therapeutic drug monitoring or specialized storage requirements. While surveyed pharmacists acknowledged these factors, they were generally viewed as manageable complications rather than major barriers. Drug–drug interactions, adverse events and administration complexities are inherent challenges with antimicrobials that specialist pharmacists routinely navigate in clinical practice. Through active medication review, dose adjustment and patient monitoring, pharmacists can substantially mitigate many of these complications, potentially minimizing the value differential quantified in the Product Award Criteria. For instance, respondents indicated that refrigerated storage of intravenous antimicrobials, penalized under the health system factors, would not meaningfully impact product value in practice. Subscription antimicrobials are used in select patient populations, typically in hospital settings with established cold-chain infrastructure and under direct specialist pharmacist supervision, thereby mitigating storage-related challenges. It should be noted, however, that the high weighting of these criteria, which address logistical factors like storage and monitoring, would be far more applicable and critical if such innovative funding models were to be deployed in settings with limited infrastructure, such as Low- and Middle-Income Countries (LMICs).

However, pharmacists identified one clear limitation that warrants greater weighting: the need for aseptic preparation. Aseptic compounding facilities are not universally available across the NHS, and dependence on aseptic preparation would significantly impede timely access to treatment, particularly in smaller or rural hospitals. This barrier could undermine equitable access to novel antimicrobials and merits serious consideration in value-based assessments.

The overall misalignment between Product Award Criteria ‘health system benefits’ measures and the perceived value assigned by this expert pharmacy audience likely reflects the experience and confidence of specialist pharmacists in managing complex dosing, monitoring and administration requirements. This divergence necessitates re-evaluation of certain quantified measures, with consideration of higher weighting for once-daily intravenous or oral formulations, reduced penalties for refrigerated storage when used in supervised hospital settings and recognition that many drug-related complexities can be effectively managed through specialist pharmacist support. Conversely, barriers that truly limit access, such as aseptic preparation requirements, should receive greater emphasis.

An important limitation of the subscription model that warrants acknowledgment is that it does not eliminate the impact of drug acquisition costs but rather reduces them through negotiated pricing. Even with substantial discounts, subscription antimicrobials remain high-cost therapies with significant financial premiums over established generic alternatives.^10^ At the Trust level, the influence of drug cost persists and may continue to limit patient access to certain treatments, contrary to the subscription model's core objective of decoupling usage from cost. Local formulary decisions, budget constraints and prescriber perceptions of cost-effectiveness may still create barriers to appropriate use, suggesting that further work is needed to fully realize the subscription model's promise of unrestricted access based on clinical need.^16^

The survey responses suggest that there is no consensus on what constitutes a fair price, with specialist pharmacists suggesting a payment of 0–75% of total cost; median 50%. This indicates that a significant proportion of participants believe that Trusts should pay half of the list price for the drug under the subscription model.

This study has several limitations that should be considered when interpreting our findings. While our respondents represent experienced practitioners actively engaged in antimicrobial stewardship, their views may not capture the full diversity of perspectives across all NHS settings (especially given most respondents were based in London), particularly smaller hospitals or those with less developed stewardship programmes. Opinions of other professional groups or tax-payer priorities might also differ. Additionally, this survey captures perceptions and opinions rather than objective outcome data, and further research examining actual prescribing patterns, patient outcomes and cost-effectiveness under the subscription model would provide valuable complementary evidence. A non-validated study questionnaire was used, although its design was informed by a previous study and was sufficiently piloted. Finally, it should be noted that this study was funded by an educational grant from a commercial company, yet the company was not involved in the conduct of the survey and interpretation of the study results. A relatively high participation incentive was offered (BSAC conference attendance fee, worth up to £200), the study responses were not anonymous and the authors of the study were supportive of subscription models for antimicrobials, which might have skewed results towards more favourable responses.

### Conclusions

The NICE subscription model is viewed positively by AMS pharmacists and has demonstrably influenced antimicrobial prescribing practices for multidrug-resistant infections since its introduction. However, our findings suggest that the Product Award Criteria used to assess future antimicrobials may require adjustment to better reflect practical clinical realities and the capabilities of specialist pharmacy services to mitigate certain drug-related complexities. We identified a strong need for subscription models to fund agents with primarily anti-Gram-negative activity with convenient dosing regimens that would allow outpatient treatment via OPAT, while maintaining acceptable monitoring and storage requirements. Ongoing evaluation, stakeholder engagement and iterative adaptation will be crucial to ensure the model's continued relevance and effectiveness in addressing the urgent challenge of AMR. As the subscription model potentially expands to include additional drug classes and other healthcare systems consider similar approaches, the insights from frontline specialists will be invaluable in shaping sustainable and clinically meaningful antimicrobial funding frameworks.

## Supplementary Material

dlag018_Supplementary_Data

## Transparency declarations

S.H. received consultant or educational support from Advanz, Baxter, BowMed, Eumedica, Gilead, NAPP, Pfizer, Shionogi and Tillotts. I. B. reports investigator-initiated research funding from Shionogi B.V and Pfizer; honoraria and travel grants from Shionogi B.V., Menarini UK and Tillotts Pharma; participating in advisory boards for GSK, InfectoPharm and Eumedica. Ordinary Council Member for the British Society for Antimicrobial Chemotherapy. M.G. received consultant and educational support from Advanz, Baxter, Eumedia, NAPP and Pfizer. Into Clinical Practice Lead for the British Society for Antimicrobial Chemotherapy.

## Supplementary data

Appendix I is available as Supplementary data at *JAC-AMR* Online.
