# Supplementary material for: Exploring the perspectives of antimicrobial stewardship pharmacists in England on the subscription model for antimicrobial drugs
Source: JAC Antimicrob Resist. 2026 Feb 17;8(1):dlag018. doi: 10.1093/jacamr/dlag018 (PMC12910370; doi:10.1093/jacamr/dlag018)
Supplement: dlag018_Supplementary_Data [file dlag018_supplementary_data.zip › SMASH II Appendix I.pdf]

BRITISH SOCIETY FOR ANTIMICROBIAL CHEMOTHERAPY

SMASH

SUBSCRIPTION MODELS FOR ANTIBIOTICS IN HOSPITALS

SMASH2 survey

Welcome to the SMASH survey (Subscription Models for AntibioticS in Hospitals).

Dear Colleague,

Thank you for considering completing the SMASH survey (Subscription Models for Antibiotics in Hospitals). This study is an opportunity for you to express your views on the implementation of a novel funding model for antimicrobials in the National Health Service (NHS) and follows on from a recent similar survey among infection consultants in England (https://academic.oup.com/jacamr/article/5/4/dlad091/7234491). We hope that the findings of this survey will influence future health policy and improve the care of patients with drug-resistant infections in the UK. The SMASH survey should take approximately 30 minutes to complete. It is sponsored by the British Society of Antimicrobial Chemotherapy (BSAC) and funded by Shionogi B.V. To thank you for your time completing this survey, you will be provided with a £200 voucher for attending a BSAC conference or course (to be sent electronically in March 2025).

Study background:

On 1st July, 2022 NHS England, in collaboration with the National Institute for Health and Care Excellence (NICE), launched a novel "subscription-type" payment model pilot. This first-of-a-kind de-linked funding model means that the NHS will pay the pharmaceutical companies producing antimicrobials drugs a fixed pre-agreed annual fee (the "subscription"), irrespective of volumes of antimicrobails actually used by individual Trusts. So, rather than paying for antimicrobials on a "number of vials used" basis, this funding model can be considered as a "subscription for access" for antimicrobial agents for the NHS as a whole. The individual NHS Trusts still pay a discounted list price for these drugs – NHS England tops up these payments received to reach the pre-agreed subscription amount. This funding model aims to align industry financial incentives with good stewardship practices, and incentivise pharmaceutical companies to bring new antimicrobial products to market. It will also allow affordable use of novel antimicrobials for patients with drug-resistant infections in individual Trusts. This initiative is part of the 2019 – 2024 and 2024 – 2029 UK Antimicrobial Resistance 5-year National Action Plans and represents the world's first fully delinked funding model for antimicrobials. After a successfull 3-year initial pilot with ceftazidime-avibactam and cefiderocol, the NHS is now establishing a permanent "subscription-type" payment model that will include existing and new antimicrobials launching in the UK market. You can find additional information on the new subscription-type payment model in the following links:

https://www.nice.org.uk/about/what-we-do/life-sciences/scientific-advice/models-for-the-evaluation-and-purchase-of-antimicrobials

https://www.england.nhs.uk/long-read/antimicrobial-products-subscription-model-guidance-on-commercial-arrangements/ https://www.nice.org.uk/about/what-we-do/life-sciences/scientific-advice/models-for-the-evaluation-and-purchase-of-antimicrobials

The SMASH survey aims to capture the views of Lead Antimicrobial Stewardship/Infection Pharmacists on the implementation and impact of the novel ‘subscription-type’ payment model in the NHS. This should allow us to collate expert opinion on important aspects of the de-linked funding models, as well as inform upcoming contract negotiations and future health policy. The SMASH questionnaire is not a test of knowledge and aims to capture participant opinions only. Survey results will be pooled and analyzed at regional level, while individual responses will be stored in password protected NHS computers, to preserve anonymity and confidentiality of study participants. At the end of the survey, you will be invited to indicate whether you would be interested in receiving the survey results. Thank you for your consideration and your time,

Dr Ioannis Baltas & Professor Mark Gilchrist

By clicking the “Next” button below, you consent to participate in this study.

BRITISH SOCIETY FOR ANTIMICROBIAL CHEMOTHERAPY

SMASH

SUBSCRIPTION MODELS FOR ANTIBIOTICS IN HOSPITALS

SMASH2 survey

Survey Eligibility

* 1. Are you the Lead Antimicrobial Stewardship/Infection Pharmacist of an NHS Acute Trust?

- ◯ Yes
- ◯ No

BRITISH SOCIETY FOR ANTIMICROBIAL CHEMOTHERAPY

SMASH

SUBSCRIPTION MODELS FOR ANTIBIOTICS IN HOSPITALS

SMASH2 survey

Demographics

* 2. Please state the year that you started working as a Pharmacist:

____

* 3. Please state the year that you started working as an Antimicrobial Stewardship/Infection Pharmacist:

____

* 4. Please indicate the NHS region you are currently working in:

- ◯ East of England
- ◯ London
- ◯ Midlands
- ◯ North East and Yorkshire
- ◯ North West
- ◯ Northern Ireland
- ◯ Scotland
- ◯ South East
- ◯ South West
- ◯ Wales

* 5. Please provide the name of the NHS Trust(s)/Health Boards you currently work in (please avoid abbreviations):

[ ]

* 6. Do you have any relevant conflicts of interest when completing this survey (this does not preclude you from completing the survey)?

- ◯ Yes
- ◯ No

BRITISH SOCIETY FOR ANTIMICROBIAL CHEMOTHERAPY

SMASH — SUBSCRIPTION MODELS FOR ANTIBIOTICS IN HOSPITALS

SMASH2 survey

The antimicrobial subscription model

* 7. Before completing this survey, had you previously heard about the ‘subscription-type’ payment model for antimicrobial drugs?

- ◯ Yes
- ◯ No

On the scale shown, how much do you agree with the following statements about the UK’s ‘subscription-type’ payment model for antimicrobial drugs?

* 8. Before completing this survey, I had a good understanding of the ‘subscription-type’ payment model for antimicrobial drugs.

- Strongly agree
- Agree
- Neither agree nor disagree
- Disagree
- Strongly disagree

* 9. At the launch of the ‘subscription-type’ payment model in July 2022 I had received adequate information about it.

- Strongly agree
- Agree
- Neither agree nor disagree
- Disagree
- Strongly disagree

* 10. The ‘subscription-type’ payment model is a welcome development in the management of drug-resistant infections.

- Strongly agree
- Agree
- Neither agree nor disagree
- Disagree
- Strongly disagree

* 11. Access to novel antimicrobials through the ‘subscription-type’ payment model will improve the ability of infection specialists to treat drug-resistant infections.

- Strongly agree
- Agree
- Neither agree nor disagree
- Disagree
- Strongly disagree

* 12. Cost is a significant consideration for me when using/recommending the use of specific antimicrobials:

- Strongly agree
- Agree
- Neither agree nor disagree
- Disagree
- Strongly disagree

* 13. The ‘subscription-type’ payment model will stimulate research and development of new antimicrobials.

- Strongly agree
- Agree
- Neither agree nor disagree
- Disagree
- Strongly disagree

* 14. The ‘subscription-type’ payment model should be expanded to include antifungal drugs:

- Strongly agree
- Agree
- Neither agree nor disagree
- Disagree
- Strongly disagree

* 15. The ‘subscription-type’ payment model should be expanded to include antiviral drugs:

- Strongly agree
- Agree
- Neither agree nor disagree
- Disagree
- Strongly disagree

* 16. The ‘subscription-type’ payment model should be expanded to include antiparasitic drugs:

- Strongly agree
- Agree
- Neither agree nor disagree
- Disagree
- Strongly disagree

* 17. The ‘subscription-type’ payment model should be expanded to include non-antimicrobials agents including monoclonal antibodies, phages, anti-virulence agents etc:

- Strongly agree
- Agree
- Neither agree nor disagree
- Disagree
- Strongly disagree

BRITISH SOCIETY FOR ANTIMICROBIAL CHEMOTHERAPY

SMASH SUBSCRIPTION MODELS FOR ANTIBIOTICS IN HOSPITALS

SMASH2 survey

STEDI values (Spectrum, Transmission, Enablement, Diversity, Insurance)

* 18. The term “insurance value” refers to the value of having antimicrobials available in case of a sudden or major increase in the prevalence of infections with pathogens resistant to all other existing antimicrobials. On a scale of 1 – 5, please assess the importance of high insurance value for candidate new antimicrobials:

1	2	3	4	5
★	★	★	★	★

1 = Low importance 3 = Medium importance 5 = High importance

* 19. The term “enablement value” refers to the benefits associated with enabling other treatments or procedures to take place, e.g., surgical and medical procedures that may not be possible if antimicrobials were not available to prevent or treat surgical site or post-procedure infections. On a scale of 1 – 5, please assess the importance of high enablement value for candidate new antimicrobials:

1	2	3	4	5
★	★	★	★	★

1 = Low importance 3 = Medium importance 5 = High importance

* 20. The term “transmission value” refers to the benefits of avoiding the spread of the pathogen to the wider population if the patient with the infection responds promptly to treatment and is treated successfully. On a scale of 1 – 5, please assess the importance of high transmission value for candidate new antimicrobials:

1	2	3	4	5
★	★	★	★	★

1 = Low importance 3 = Medium importance 5 = High importance

* 21. The term “diversity value” refers to the benefits of having a range of treatment options available to reduce selection pressure for resistance and to preserve the efficacy of existing antimicrobials. For example, a new antimicrobial, typically effective against Vancomycin-resistant Staphylococcus aureus, where alternative treatment options are limited, would have high diversity value, while a new antimicrobial typically effective against Group A streptococcus, where multiple alternative treatment options are available, would have low diversity value. On a scale of 1 – 5, please assess the importance of high diversity value for candidate new antimicrobials:

1	2	3	4	5
☆	☆	☆	☆	☆

1 = Low importance 3 = Medium importance 5 = High importance

* 22. The term “spectrum value” refers to the benefits of replacing other broad-spectrum antimicrobials that could be used to cure the same infection, with a narrower spectrum antimicrobial in order to preserve the patient’s microbiome. For example, an antimicrobial with a spectrum similar to flucloxacillin would be considered to have high spectrum value, while an antimicrobial with a spectrum similar to piperacillin/tazobactam would be considered to have low spectrum value. On a scale of 1 – 5, please assess the importance of high spectrum value for candidate new antimicrobials:

1	2	3	4	5
☆	☆	☆	☆	☆

1 = Low importance 3 = Medium importance 5 = High importance

BRITISH SOCIETY FOR ANTIMICROBIAL CHEMOTHERAPY

SMASH — SUBSCRIPTION MODELS FOR ANTIBIOTICS IN HOSPITALS

SMASH2 survey

Antimicrobials in the subscription-type model

* 23. New antimicrobials introduced through the ‘subscription-type’ payment model should be chosen based on their activity against the WHO priority pathogen list.

- ◯ Strongly agree
- ◯ Agree
- ◯ Neither agree nor disagree
- ◯ Disagree
- ◯ Strongly disagree

* 24. On a scale of 1 – 5, please assess the degree of unmet need for effective treatments for the following pathogens in your Trust.

1 = low unmet need 3 = moderate unmet need 5 = high unmet need

	1	2	3	4	5
Enterobacterales, 3rd generation cephalosporin-resistant, ESBL-producing	○	○	○	○	○
Enterobacterales, 3rd generation cephalosporin-resistant, AmpC-producing	○	○	○	○	○
Enterobacterales, carbapenem-resistant	○	○	○	○	○
Enterobacterales, OXA-48-producing	○	○	○	○	○
Enterobacterales, MBL-producing	○	○	○	○	○
Enterobacterales, KPC-producing	○	○	○	○	○
Pseudomonas aeruginosa, carbapenem-resistant	○	○	○	○	○
Acinetobacter baumannii, carbapenem-resistant	○	○	○	○	○
Neisseria gonorrhoeae, 3rd Generation cephalosporin-resistant	○	○	○	○	○
Enterococcus faecium, vancomycin-resistant	○	○	○	○	○
Staphylococcus aureus, methicillin-resistant	○	○	○	○	○
Campylobacter species, fluoroquinolone-resistant	○	○	○	○	○
Salmonella species, fluoroquinolone-resistant	○	○	○	○	○
Shigella species, fluroquinolone-resistant	○	○	○	○	○
Helicobacter pylori, clarithromycin-resistant	○	○	○	○	○
Streptococcus pneumoniae, penicillin-non-susceptible	○	○	○	○	○

Haemophilus influenzae, ampicillin-resistant					

* 25. Off-license treatment of paediatric patients (<18 years old) with infections due to multidrug-resistant bacteria using newly licensed antimicrobials should be considered.

- Strongly agree
- Agree
- Neither agree nor disagree
- Disagree
- Strongly disagree

* 26. Off-license treatment of pregnant patients with infections due to multidrug-resistant bacteria using newly licensed antimicrobials should be considered.

- Strongly agree
- Agree
- Neither agree nor disagree
- Disagree
- Strongly disagree

* 27. Need for therapeutic drug monitoring would be a significant downside for antimicrobials introduced through the subscription-type’ payment model.

- Strongly agree
- Agree
- Neither agree nor disagree
- Disagree
- Strongly disagree

* 28. Significant drug-drug interactions that require dose or timing adjustment would be a significant downside for antimicrobials introduced through the subscription-type’ payment model.

- Strongly agree
- Agree
- Neither agree nor disagree
- Disagree
- Strongly disagree

* 29. Requirement for fridge storage would be a significant downside for antimicrobials introduced through the subscription-type’ payment model:

- Strongly agree
- Agree
- Neither agree nor disagree
- Disagree
- Strongly disagree

* 30. Requirement for reconstitution would be a significant downside for antimicrobials introduced through the subscription-type’ payment model.

- Strongly agree
- Agree
- Neither agree nor disagree
- Disagree
- Strongly disagree

* 31. Requirement for aseptic services would be a significant downside for antimicrobials introduced through the subscription-type’ payment model.

- Strongly agree
- Agree
- Neither agree nor disagree
- Disagree
- Strongly disagree

BRITISH SOCIETY FOR ANTIMICROBIAL CHEMOTHERAPY

SMASH
SUBSCRIPTION MODELS FOR ANTIBIOTICS IN HOSPITALS

SMASH2 survey

OPAT

* 32. Suitability for administration through Outpatient Parenteral Antimicrobial Therapy (OPAT) services is a desirable characteristic of antimicrobials introduced through the subscription-type’ payment model.

- Strongly agree
- Agree
- Neither agree nor disagree
- Disagree
- Strongly disagree

* 33. On a scale of 1 – 5, please assess the desirability of the following routes and frequencies of administration for antimicrobials introduced through the subscription-type' payment model.

1 = Not desirable 3 = desirable 5 = Highly desirable

	1	2	3	4	5
Inhalational or nebulised	○	○	○	○	○
Intravenous, single or once weekly	○	○	○	○	○
Oral	○	○	○	○	○
Intravenous, once a day	○	○	○	○	○
Intravenous, twice a day	○	○	○	○	○
Intravenous, three times a day	○	○	○	○	○
Intravenous, four times a day	○	○	○	○	○
Intravenous, continuous infusion	○	○	○	○	○

* 34. Antimicrobials introduced through the 'subscription-type' payment model that are suitable for OPAT are likely to be associated with decreased length of stay in hospital.

- ○ Strongly agree
- ○ Agree
- ○ Neither agree nor disagree
- ○ Disagree
- ○ Strongly disagree

BRITISH SOCIETY FOR ANTIMICROBIAL CHEMOTHERAPY

SMASH

SUBSCRIPTION MODELS FOR ANTIBIOTICS IN HOSPITALS

SMASH2 survey

Antimicrobial stewardship

* 35. The ‘subscription-type’ payment model will lead to reduced use of older broad-spectrum agents.

- Strongly agree
- Agree
- Neither agree nor disagree
- Disagree
- Strongly disagree

* 36. Outcomes of patients receiving antimicrobials through the ‘subscription-type’ payment model are being monitored through the UK Antimicrobial Registry, currently being developed by the British Society of Antimicrobial Chemotherapy. Registration of patients is currently voluntary.

A national registry is necessary to monitor outcomes of patients, who receive antimicrobials through the ‘subscription-type’ payment model.

- Strongly agree
- Agree
- Neither agree nor disagree
- Disagree
- Strongly disagree

* 37. Prescribing indications for antimicrobials introduced through the ‘subscription-type’ payment model is being monitored through the use of Blueteq forms, which is currently voluntary.

Completion of a Blueteq form during drug dispensing is necessary for ensuring stewardship of the prescribing of antimicrobials provided through the ‘subscription-type’ payment model.

- Strongly agree
- Agree
- Neither agree nor disagree
- Disagree
- Strongly disagree

* 38. Completion of a prescribing authorization process (such as a Blueteq form) during dispensing of antimicrobials introduced through the ‘subscription-type’ payment model should be the responsibility of which of the following professionals (select all that apply)?

- [ ] Infectious Diseases or Microbiology Consultant
- [ ] Infectious Diseases or Microbiology Registrar
- [ ] Antimicrobial stewardship pharmacist
- [ ] Ward pharmacist
- [ ] Clinical team in charge of patient’s care
- [ ] Other (please specify)

  ____

- [ ] Completion of a prescribing authorization process should not be necessary

* 39. In your opinion, please select the **minimum** pre-authorization requirement for the prescription of antimicrobials introduced through the ‘subscription-type’ payment model.

Antimicrobials introduced through the ‘subscription-type’ payment model can be prescribed:

- ○ By the clinical teams, without consultation from an infection specialist.
- ○ After recommendation from the infectious diseases or microbiology registrar, without the opinion of the infectious diseases or microbiology consultant.
- ○ After recommendation from a single infectious diseases or microbiology consultant, without the opinion of an antimicrobial stewardship pharmacist.
- ○ After recommendation from a single infectious diseases or microbiology consultant and a single antimicrobial stewardship pharmacist.
- ○ After discussion in a multidisciplinary meeting, involving consensus of multiple infectious diseases or microbiology consultants and an antimicrobial stewardship pharmacist.

* 40. In your opinion, antimicrobials introduced through the ‘subscription-type’ payment model should be available as a treatment option for which of following hierarchical scenarios?

- ○ Empirically, when there is urgent clinical need to treat an unwell patient with a severe infection, in the absence of risk factors for antimicrobial resistance, and all the scenarios below.
- ○ Empirically, when there is urgent clinical need to treat an unwell patient with a severe infection, when risk factors for antimicrobial resistance are present, and all the scenarios below.
- ○ In microbiologically confirmed carbapenem-resistant infections, when microbiological susceptibility and/or genetic testing for the proposed antimicrobial is unknown, and the scenario below.
- ○ In microbiologically confirmed carbapenem-resistant infections, when microbiological susceptibility and/or genetic testing has confirmed that the infection is susceptible to the proposed antimicrobial.

* 41. In your opinion, which of the following scenarios would justify empirical use of antimicrobials introduced through the ‘subscription-type’ payment model in an unwell patient with a severe infection (select all that apply)?

- [ ] Current colonization with carbapenem-resistant bacteria
- [ ] Previous infection with carbapenem-resistant bacteria
- [ ] Clinical treatment failure of broad-spectrum non-carbapenem antibiotics
- [ ] Clinical treatment failure of carbapenem antibiotics
- [ ] Ward outbreak of carbapenem-resistant bacteria
- [ ] Recent admission to a high prevalence hospital for carbapenem-resistant bacteria (in England or abroad)
- [ ] Significant prior broad-spectrum antibiotic exposure (excluding carbapenems)
- [ ] Significant prior exposure to carbapenems
- [ ] Significant prior healthcare exposure (e.g. multiple recent admissions with prolonged length of stay)
- [ ] Recent surgery or invasive procedure
- [ ] Current or recent admission to Intensive Care
- [ ] None of the above

* 42. In patients with stage IV (severe) or V (end-stage) chronic kidney disease and infections caused by multi-drug resistant bacteria, new antimicrobials are preferable to colistin, aminoglycosides or glycopeptides as initial treatment options due to the risks of nephrotoxicity, despite the antimicrobial stewardship need to restrict the use of antimicrobials introduced through the ‘subscription-type’ payment model.

- ○ Strongly agree
- ○ Agree
- ○ Neither agree nor disagree
- ○ Disagree
- ○ Strongly disagree

* 43. In your opinion, please indicate how effective the following interventions are for the antimicrobial stewardship of antimicrobials introduced through the ‘subscription-type’ payment model in a scale of 1 – 5.

(1 = Minimally effective, 5 = Very effective)

	1	2	3	4	5
Education of infection specialists	◯	◯	◯	◯	◯
Education of clinical teams (outside of infection services)	◯	◯	◯	◯	◯
Local guidelines on patient eligibility criteria	◯	◯	◯	◯	◯
National guidelines on patient eligibility criteria	◯	◯	◯	◯	◯
Local audit cycle of appropriateness of prescribing	◯	◯	◯	◯	◯
Antimicrobial stewardship ward-rounds	◯	◯	◯	◯	◯
Electronic prescribing systems with prompts/alerts/reminders	◯	◯	◯	◯	◯
Electronic prescribing systems with controls/automatic stop orders	◯	◯	◯	◯	◯
Individualized prescriber feedback	◯	◯	◯	◯	◯
Requirement for pre-authorization by infection specialist	◯	◯	◯	◯	◯
The presence of an antimicrobial pharmacy team	◯	◯	◯	◯	◯

* 44. The “subscription-type” payment model for antimicrobial drugs requires that individual hospital Trusts still pay a heavily discounted list price for new antimicrobials introduced through the model according to volumes used, while the NHS tops up the remaining difference to the pre-agreed subscription amount. This model aims to strike the right balance between low enough price for drugs to be affordable where clinically indicated and high enough to avoid unjustified use. Under this model, what would you consider to be a fair price for individual Trusts to pay for a hypothetical drug with a list price of £1000? For example, a Trust price of £500 would mean that, for every course of the drug the Trust would pay £500 and NHS England would top up another £500. Please put a whole number between 0 and 1000 in the text box below indicating the Trust price you consider fair.

BRITISH SOCIETY FOR ANTIMICROBIAL CHEMOTHERAPY

SMASH

SUBSCRIPTION MODELS FOR ANTIBIOTICS IN HOSPITALS

SMASH2 survey

Survey finish and contact details

* 45. Would you like to receive the written report of this survey?

- ◯ Yes
- ◯ No

* 46. Please provide your NHS email address below. This will only be used to ensure that only NHS healthcare professionals completed this questionnaire, send you your voucher and to send you the survey results, if requested. Your email address will not be used for any other purposes including sending you spam emails and will not be stored after the end of this study.

Email Address ____________________

Thank you for completing the SMASH survey!

BRITISH SOCIETY FOR ANTIMICROBIAL CHEMOTHERAPY

SMASH

SUBSCRIPTION MODELS FOR ANTIBIOTICS IN HOSPITALS
