# Supplementary material for: Exploring the perspectives of antimicrobial stewardship pharmacists in England on the subscription model for antimicrobial drugs
Source: JAC Antimicrob Resist. 2026 Feb 17;8(1):dlag018. doi: 10.1093/jacamr/dlag018 (PMC12910370; doi:10.1093/jacamr/dlag018)
Supplement: dlag018_Supplementary_Data [file dlag018_supplementary_data.zip › SMASH II Supplementary materials.docx]

Supplementary Figure 1: Distribution of survey responses by NHS region within England, UK.
